# On Modern Progress in Ophthalmic Medicine and Surgery

**Published:** 1895-05-04

**Authors:** Robert Brudenell Carter

**Affiliations:** Consulting Ophthalmic Surgeon to St. George's Hospital


### On Modern Progress in Ophthalmic Medicine and Surgery.

By Robert Brudenell Carter, F.R.C.S., Consulting Ophthalmic Surgeon to St. George's Hospital.

### DISEASES OF THE CORNEA.

(*Continued from page 21*).

The tendency to healing which, in a sloughing ulcer of the cornea, is generally an immediate consequence of the diminished ocular tension incidental to perforation, has for a long period been utilised by surgeons. Many years ago Saemisch proposed to divide such an ulcer through the base by transfixing the cornea with a narrow cataract knife, entered and brought out in the sound tissue on either side of the diseased portion; and he advised that the edges of the incision thus made should be daily separated by means of a probe until a definite reparative process had commenced. The method was temporarily effective, but was almost always followed by adhesion of the iris to the cicatrix of the incision, and left the eye exposed to all the subsequent risks which such an adhesion would entail. Frequently, moreover, the scar of the combined ulcer and incision was so situate as to be a lasting impediment to vision.

Assuming an ulcer not to be neuro-paralytic, but to owe its sloughing tendency to microbial infection, the first considerations should be whether its depth is too great to admit of satisfactory scraping, and whether its position is such that its cicatrix must necessarily cover the pupil and interfere with sight. If the ulcer be not very deep, its surface should be completely removed by a small, sharp spoon, or destroyed by the galvanic cautery, the new surface freely irrigated with some antiseptic solution, and the eyelids closed with compress and plaster. The dressing may be left undisturbed for forty-eight hours, and, when the eye is opened for inspection, it should again be irrigated before being closed. If matters are progressing favourably, this treatment may be sufficient, and the question of an artificial pupil may be left for future consideration, when the size, character, and position of the cicatrix are determined. If, at the first dressing, the ulcer is found generally healthy, but extending or threatening to extend in some particular direction, the unhealthy locality may be scraped or cauterised again, and the closure renewed; but if the appearance of the whole or of the greater part be unsatisfactory there should be no delay in the performance of iridectomy. In selecting the portion of the iris to be removed, the surgeon should consider in what position the resulting coloboma will be most useful as an artificial pupil to give vision by the side of the ultimate cicatrix, and hence he must endeavour to operate behind clear cornea, and in a position where the opening will not be covered by the upper lid. When the state of the cornea permits, the best position is downwards and inwards, the next best directly downwards, and the directly inward and directly outward positions are less useful. Before the operation the eye should receive a free application of a solution of eserine, which has a marked effect in arresting the migration of leucocytes from the diseased into the still healthy tissue; and closure for forty-eight hours may be maintained after the operation has been effected. The iridectomy will usually be followed by uninterrupted healing, and the extent of the cicatricial flattening of the cornea will depend upon the size and depth which the ulcer had attained before the operation was performed. If these are considerable when the case is first seen, no time should be lost in the employment of less effectual methods.

In any case of sloughing ulcer not sufficiently large or important to call for immediate iridectomy, I should now supplement the processes of scraping or cauterisation by the sub-conjunctival injection of a solution of perchloride of mercury, a method of treatment introduced into practice by Darier, and which has been largely used in a great variety of affections, both acute and chronic, in many of which it seems little likely to be attended with advantage. The efficacy, and indeed the safety, of the little operation appear to depend upon the strict observance of antiseptic precautions; and, on this account, it is necessary to be provided with a syringe which admits of being completely sterilised, and with iridium-pointed platinum needles, which can be heated in a lamp flame immediately prior to use. A solution of 1 in 1,000 is found to be of convenient strength, and of this about three or four minims may be injected at a time. The eye should be thoroughly cocainised, a fold of conjunctiva pinched up with forceps, and the point of the needle pushed into the sub-conjunctival tissue, in a direction away from the cornea, at any part of the circumference. The subsequent pain or smarting is usually quite trivial; and, in conditions of acute disease, such as some forms of inflammation, the injection may be repeated daily, or even twice a day, while in chronic maladies longer intervals are desirable. If cocaine wafers are employed as a preliminary application, all trace of the gelatine entering into their composition should be removed by washing with an antiseptic liquid before the puncture is made, but the necessity for this may be avoided by using a sterilised cocaine solution, containing also a minute quantity of salicylic acid. Cocaine hydrochlorate 10 parts, distilled water 90, salicylic acid 0.01, will be found a good proportion for the purpose; while as an antiseptic wash I prefer either a 15 per cent. solution of Barff's boroglyceride, or a preparation recently introduced under the name of "Izal," which is said to be a most effective bactericide, and which is certainly absolutely unirritating to the conjunctiva or cornea. My attention was called to this preparation some months ago, by the published experiences of Mr. Bruce Clarke, and I have since employed it in eye operations with exceedingly satisfactory results. It has no deleterious action upon instruments, and, as a teaspoonful of the liquid as sold is sufficient for a pint of water, it has the incidental advantage of being extremely portable. I have heard that there is some doubt as to the perfect uniformity of the preparation, but that this question is engaging the attention of chemists, and that the doubt, if such there be, is likely soon to be removed. As far as my own experience goes, I cannot but consider the new antiseptic to be a welcome addition to the resources of the ophthalmic surgeon. Izal is of near kindred to carbolic acid, and seems both to possess its powers and to be free from many of its disadvantages.

